# Diabetes and Pre-Diabetes as Determined by Glycated Haemoglobin A1c and Glucose Levels in a Developing Southern Chinese Population

**DOI:** 10.1371/journal.pone.0037260

**Published:** 2012-05-15

**Authors:** Yong Hui Zhang, Wen Jun Ma, G. Neil Thomas, Yan Jun Xu, Xiang Qian Lao, Xiao Jun Xu, Xiu Ling Song, Hao Feng Xu, Qiu Mao Cai, Liang Xia, Shao Ping Nie, Hui Hong Deng, Ignatius Tak Sun Yu

**Affiliations:** 1 Centre for Disease Control and Prevention of Guangdong Province, Guangzhou, China; 2 Guangdong Institute of Public Health, Guangzhou, China; 3 School of Health and Population Sciences, the University of Birmingham Edgbaston, United Kingdom; 4 School of Public Health and Primary Care, Faculty of Medicine, the Chinese University of Hong Kong, Hong Kong, China; 5 The Chinese University of Hong Kong Shenzhen Research Institute, Shenzhen, China; Johns Hopkins Bloomberg School of Public Health, United States of America

## Abstract

**Background:**

The American Diabetes Association and World Health Organization have recently adopted the HbA1c measurement as one method of diagnostic criteria for diabetes. The change in diagnostic criteria has important implications for diabetes treatment and prevention. We therefore investigate diabetes using HbA1c and glucose criteria together, and assess the prevalent trend in a developing southern Chinese population with 85 million residents.

**Methods:**

A stratified multistage random sampling method was applied and a representative sample of 3590 residents 18 years of age or above was obtained in 2010. Each participant received a full medical check-up, including measurement of fasting plasma glucose, 2-hour post-load plasma glucose, and HbA1c. Information on history of diagnosis and treatment of diabetes was collected. The prevalence of diabetes obtained from the present survey was compared with the data from the survey in 2002.

**Results:**

The prevalence of diabetes based on both glucose and HbA1c measurements was 21.7% (95% CI: 17.4%–26.1%) in 2010, which suggests that more than 1 in 5 adult residents were suffering from diabetes in this developing population. Only 12.9% (95% CI: 8.3%–17.6%) of diabetic residents were aware of their condition. The prevalence of pre-diabetes was 66.3% (95% CI: 62.7%–69.8%). The prevalence of diabetes and pre-diabetes which met all the three diagnostic thresholds (fast plasma glucose, 2 hour post-load plasma glucose, and HbA1c) was 3.1% and 5.2%, respectively. Diabetes and pre-diabetes as determined by HbA1c measurement had higher vascular risk than those determined by glucose levels. The prevalence of diabetes increased from 2.9% (95% CI: 2.0%–3.7%) in 2002 to 13.8% (95% CI: 10.2%–17.3%) in 2010 based on the same glucose criteria.

**Conclusions:**

Our results show that the diabetes epidemic is accelerating in China. The awareness of diabetes is extremely low. The glucose test and HbA1c measurement should be used together to increase detection of diabetes and pre-diabetes.

## Introduction

The fasting plasma glucose (FPG) test and the oral glucose tolerance test (OGTT) are the most common methods for screening and diagnosing diabetes. It has been debated for several decades whether the glycated haemoglobin A1c (HbA1c) test, a standard monitoring test for the control of diabetes, could be used to diagnose diabetes. HbA1c level serves as a biomarker to capture longer-term glycaemic exposure over time and is more intimately related to the risk of complications than a single or episodic measurement of glucose levels [1]. Concurrent with the improvement and standardisation of the glycated haemoglobin A1c (HbA1c) assay, an International Expert Committee in 2009 recommended that diabetes could be diagnosed when the HbA1c level is more than 6.5%, provided it is measured using the standardised assay method [1]. The American Diabetes Association (ADA) subsequently adopted the use of the HbA1c measurement as one method of diagnostic criteria in 2010 [2], as did the World Health Organization (WHO) in 2011 [3].

The change in diagnostic criteria has important clinical and public health implications. The inclusion of HbA1c will increase the feasibility and dissemination of diabetes screening because it eliminates the need for fasting before testing. A large number of people previously considered to be healthy would be reclassified as diabetic [4]. In China, several large-scale surveys on diabetes have been conducted [5], [6], [7], [8]. The results show that the prevalence of diabetes has increased markedly in last two decades. It is thought that the rapid economic development and the aging population in China have contributed to this increased prevalence. The latest nationwide survey by Yang et al. in 2007 showed that the prevalence of diabetes in China had reached 9.7% [7]. However, all the previous surveys did not include the HbA1c measurement, which means they may have overlooked a substantial part of the population with diabetes or at high risk for diabetes and cardiovascular disease.

Guangdong is a province located in southern China with a population of 85 million residents [9]. The capital is Canton, which is approximately 180 kilometres north of Hong Kong. Guangdong was the first province where the Chinese leader Deng Xiao Ping started economic reform and open policy in 1979. When compared with other inland provinces, Guangdong is more economically developed and urbanised. The diabetes epidemic in Guangdong may be more pronounced and may have emerged because of the accompanying economic development and lifestyle transition. Data about diabetes in this province may provide information to aid in the development of disease prevention and intervention programmes for the inland provinces of China and other areas with rapid economic development.

We conducted the Guangdong Health Survey 2010 (GHS 2010) to investigate diabetes and pre-diabetes using the glucose and HbA1c criteria together. To assess the prevalent trend of diabetes in this developing population, we compared the data we obtained in this survey with the data from the Guangdong Nutrition and Health Survey 2002 (GNHS 2002) [10], [11].

## Methods

The GHS 2010 was conducted by the Guangdong Province Centre for Disease Control and Prevention (CDC) from October to December, 2010. Approval was obtained from the Ethics Committee of the China Centre for Disease Control. All participants gave written informed consent prior to the survey. Stratified multistage cluster sampling with probability proportional to size method was used for sampling in this survey. Briefly, the cities and counties of the province were categorised into four strata (large cities, small to medium cities, class 1 and class 2 rural areas) based on their levels of economic development as identified by the central government of China. The first stage of systematic sampling was conducted in each stratum. One district from the large cities, two districts from the small to medium cities, two counties from the class 1 rural areas and one counties from the class 2 rural counties were randomly selected based on population size. The second stage of sampling was subsequently conducted in each of the selected districts or counties: four neighbourhoods (urban) or townships (rural) were sampled from each of the selected districts or counties using the same systematic random sampling methods as that in the first stage. In the third stage, three residential committees (urban) or villages (rural) were sampled from each of the selected neighbourhood or townships using the same systematic random sampling methods as those in the first and second stages. In the fourth stage, all the households in the sampled residential committees (urban) or villages (rural) were grouped into clusters containing at least 50 households geographically, with one of the clusters randomly selected. In the fifth stage, 50 households were randomly sampled from each selected cluster, and one resident 18 years of age or above from each sampled household was selected with the Kish Grid method. If there were no residents 18 years of age or above in the selected household or the selected resident did not agree to participate in the survey, the household was replaced with a household nearby (526 [14.7%] households were replaced). Therefore, a total of 3600 households with 3600 residents aged 18 years or above were sampled in the GHS 2010. Among those, we collected complete data on 3590 participants, which were included in the present analysis.

A central survey site was set up in each selected cluster, and the participants were interviewed and received health examinations on-site. All interviews and examinations were conducted following standard protocols by physicians who received training specifically for the GHS 2010. The questionnaire collected a wide range of information including demographic, lifestyle, and family characteristics and personal disease histories. Information related to the history of the diagnosis and treatment of diabetes was also collected in the interview. Weight and height measurements of the participants were taken with light indoor clothing and without shoes. Body mass index (BMI) was calculated as weight in kilogrammes divided by the square of height in metres. The blood pressure measurement was taken in accordance with the 1999 World Health Organization/International Society of Hypertension guidelines on hypertension [12]. Three consecutive readings of blood pressure were taken using the right arms of participants after the participant rested in a seated position for 5 minutes. The average of the 2 final readings was used for analysis.

Venous blood samples were drawn in the morning after an overnight fast using vacutainer tubes. After the fasting blood sample was drawn, each participant had a 75 g oral glucose tolerance test. The FPG and the 2-hour post-load plasma glucose (2-h PG) were measured using a spectrophotometer 721/722 with the glucose oxidase method. The finger-stick capillary whole blood sample was collected using the Haemoglobin Capillary Collection System (HCCS) and shipped on cold packs to the Shanghai Institute of Endocrine and Metabolic Diseases in Shanghai, China, where the laboratory has been certified by National Glycohemoglobin Standardization Program (NGSP) for HbA1c measurement [13]. Additional plasma samples were stored in airtight tubes at −80°C prior to shipment on dry ice to the national test centre (Shanghai Ruijin Hospital) for the measurement of the lipids and insulin.

Diabetes and pre-diabetes in the present study were defined based on the criteria recommended by the ADA [14]. Previous diagnosis of diabetes was defined as self-reported doctor-diagnosed diabetes in the interview. New diagnosis of diabetes was given if the participant had the following test results: 1) they had a FPG of ≥7.0 mmol/l, 2) they had a 2-h PG of ≥11.1 mmol/l, or 3) they had an HbA1c level of ≥6.5%. Diabetes was confirmed if the participants were diagnosed with diabetes if any two out of the three tests (the FPG, the 2-h PG and the HbA1c) were both above the diagnostic thresholds. Pre-diabetes was defined if the participants had any following conditions: 1) a FPG between 5.6 to 6.9 mmol/l (Impaired Fasting Glucose [IFG]), 2) a 2-h PG between 7.8 to 11.0 mmol/l (Impaired Glucose Tolerance [IGT]), or 3) an HbA1c level between 5.7 to 6.4%.

To ascertain the level of change in the diagnoses of diabetes in this developing population, we compared the present study with GNHS 2002 [10], [11], [15]. The GNHS 2002 corresponded with the China National Nutrition and Health Survey 2002 (NNHS 2002), and the details have been described elsewhere [8], [11], [15], [16]. The GNHS 2002 used a similar sampling method and recruited a total of 6713 participants 18 years of age or above [11], [15]. The FPG test was performed for all participants, and the 2-h PG test was only performed for those who had an IFG [11]. The glucose oxidase method was used for the glucose measurement, which is the same method used in the GHS 2010. The HbA1c levels were not measured in the GNHS 2002.

The GHS 2010 and the GNHS 2002 adopted a stratified multistage cluster sampling design including all men and women 18 years of age or above. All the data analyses were performed using SAS software, version 9.2 (SAS Institute, Cary, NC, USA). In a manner similar to our previous survey analysis for the U.S. National Health and Nutrition Examination Survey (NHANES) and GNHS 2002 [11], [15], [17], the survey design parameters, including weight, stratum and cluster, were incorporated into all the analyses. The weight was derived from the 2000 census data and the associated administrative data. The non-response information was also incorporated into the weight parameter. PROC SURVEYMEANS and PROC SURVEYFREQ were used for the calculation of the means and the prevalence. The means and the prevalence calculated in this study correspond to the overall estimates for the population of people 18 years of age or above in the Guangdong province. PROC SURVEYREG and PROC SURVEYLOGISTIC were used to assess the differences between categories. Two-sided *p* values of less than 0.05 were considered statistically significant. The 95% confidence intervals were calculated and presented in the present study. The domain statement was used for the subpopulation analyses.

## Results

The mean age of the population was 50.1 (42.3, 58.0). Table 1 shows the general demographic and vascular characteristics of the populations stratified by sex and region. Women had significant lower blood pressure levels, triglyceride levels, education levels, smoking rates, and rates of alcohol consumption and higher HDL levels than men (all *p*<0.05). The rural population had a significantly higher prevalence of smoking than the urban population (*p*<0.001).

**Table 1:** Demographic and vascular characteristics by area and gender in the southern Chinese adults 18 years of age or above, 2010.

	Urban region			Rural region		
	Men (n = 786)	Women (n = 1005)	*p*	Men (n = 835)	Women (n = 964)	*p*
Age (years)	51.9 (44.3, 59.5)	50.5 (42. 9, 58.2)	0.4	50.1 (35.3, 64.8)	49.0 (39.0, 59.0)	0.0083
Age group (years)						
18–39	31.7 (28.3, 35.1)	32.4 (31.7, 33.1)	0.34	31.5 (26.0, 37.1)	32.3 (31.3, 33.2)	0.56
40–59	50.3 (49.8, 50.8)	50.2 (50.2, 50.2)	0.56	50.2 (50.0, 50.3)	49.5 (46.4,52.5)	0.38
60 or above	69.3 (65.0, 73.6)	68.9 (65.1, 72.7)	0.21	67.6 (65.5, 69.8)	68.0 (67.0, 68.9)	0.33
Body mass index (kg/m^2^)	23.4 (21.2, 25.6)	23.3 (21.4, 25.2)	0.46	22.8 (22.8, 22.9)	22.5 (21.2, 23.8)	0.37
Waist circumference (cm)	82.5 (75.2, 89.7)	78.0 (74.9, 81.1)	0.021	81.6 (77.4, 85.7)	78.2 (66.8, 89.7)	0.15
Fasting plasma glucose (mmol/L)	6.4 (5.5,7.3)	6.3 (5.7, 6.8)	0.18	5.8 (5.1, 6.5)	5.8 (4.7, 6.9)	0.66
2-hour post-load plasma glucose (mmol/L)	6.9 (6.9, 6.9)	6.9 (6.4, 7.5)	0.81	7.0 (6.2, 7.9)	7.0 (6.8, 7.1)	0.69
Fasting insulin (mmol/L)*	5.5 (4.6, 6.6)	6.2 (4.6, 8.3)	0.02	5.2 (3.1, 8.6)	5.6 (3.9, 8.1)	0.11
2-hour post-load insulin (mmol/L)*	21.3 (18.9, 24.1)	26.9 (25.5, 28.4)	0.0024	21.8 (4.8, 99.5)	23.3 (8.6,63.3)	0.63
HOMA-IR levels*	1.52 (1.13, 2.06)	1.69 (1.17, 2.46)	0.015	1.29 (0.91, 1.85)	1.40 (1.14, 1.71)	0.13
HbA1c levels (%)	6.1 (5.7, 6.5)	6.0 (5.8, 6.2)	0.45	6.0 (6.0,6.1)	6.0 (5.6, 6.3)	0.47
Systolic blood pressure (mmHg)	135 (132, 138)	128 (128, 129)	0.0026	133 (122, 144)	128 (119, 137)	0.0016
Diastolic blood pressure (mmHg)	82 (79, 86)	80 (79, 80)	0.032	81 (78, 85)	79 (77, 81)	0.0089
Total cholesterol (mmol/L)	4.3 (4.0, 4.5)	4.5 (4.1, 4.9)	0.12	4.2 (4.1, 4.2)	4.2 (4.0, 4.3)	0.72
Triglyceride (mmol/L)	1.5 (1.3, 1.7)	1.2 (0.7, 1.7)	0.033	1.5 (1.2, 1.7)	1.2 (1.0, 1.3)	0.048
HDL-cholesterol (mmol/L)	0.97 (0.77, 1.17)	1.14 (1.01, 1.28)	0.015	0.99 (0.95, 1.02)	1.08 (1.02, 1.13)	<0.001
LDL-cholesterol (mmol/L)	2.49 (2.38, 2.61)	2.61 (2.34, 2.88)	0.27	2.37 (2.33, 2.40)	2.36 (2.30, 2.43)	0.85
Education (%, <primary school)	27.1 (0.0, 55.1)	34.9 (15.5,54.3)	0.0052	32.1 (0.0, 69.9)	46.0 (21.7, 70.3)	<0.001
Smoking rates (%)	45.1 (37.0, 53.2)	1.6 (0.0, 3.3)	<0.001	51.9 (47.9, 55.9)	0.6 (0.6, 0.6)	<0.001
Alcohol consumption rates (%)	47.8 (44.2, 51.3)	22.2 (21.8, 22.7)	<0.001	46.1 (28.1, 64.2)	18.6 (3.5, 33.7)	<0.001

* Geometric means.

Table 2 shows the prevalence of normal healthy people, those with pre-diabetes and those with diabetes in this southern Chinese population stratified by sex, area and age. For healthy residents, older age was significantly associated with a lower prevalence of healthy residents (*p* = 0.004). Men had a lower prevalence of healthy residents than women (*p*<0.001). There was no significant difference between people from urban regions and people from rural regions (*p* = 0.39).

**Table 2:** The prevalence of normal healthy people, people with pre-diabetes and people with diabetes stratified by age, sex and area in the southern Chinese adults 18 years of age or above, 2010.

Category		Total	Age group (years)		
			18–39	40–59	60 or above
**Healthy residents** * **(n = 452)**					
All		12.0 (4.1, 19.9)	16.0 (81.5, 86.6)	11.6 (3.2, 19.9)	9.0 (0.0, 18.2)
Sex:	Men	9.8 (2.4, 17.2)	12.7 (11.4, 14.1)	9.1 (0.5, 17.7)	8.6 (0.2, 17.1)
	Women	13.7 (5.2, 22.3)	18.3 (12.7, 23.8)	13.4 (5.3, 21.5)	9.4 (0.0, 19.2)
Area:	Urban	10.3 (1.7, 18.8)	18.0 (16.7,19.2)	9.0 (0.0,21.9)	6.6 (0.0, 18.6)
	Rural	13.2 (1.3, 25.1)	14.8 (9.2,20.5)	13.5 (2.1, 24.7)	10.7 (0.0, 26.1)
**Pre-diabetic residents (n = 2385)**					
All		66.3 (62.7, 69.8)	67.7 (59.1, 76.4)	67.4 (65.2, 69.6)	62.3 (53.6, 71.0)
Sex:	Men	66.2 (62.3, 70.2)	65.8 (55.0, 76.5)	68.0 (64.9, 71.1)	63.3 (49.7, 76.9)
	Women	66.3 (62.1, 70.5)	69.2 (54.2, 84.2)	67.0 (64.2, 69.8)	61.4 (52.4, 70.4)
Area:	Urban	64.5 (59.6, 69.4)	66.3 (47.0, 85.7)	66.3 (61.4, 71.2)	59.0 (44.5, 73.5)
	Rural	67.5 (62.6, 72.4)	68.5 (58.6, 78.4)	68.2 (66.9,69.5)	64.7 (53.9, 75.4)
**Diabetic residents**					
**Diagnosed (n = 99)**					
All		2.8 (1.3,4.3)	1.1 (0.0, 3.8)	2.2 (1.9, 2.6)	5.8 (4.0, 7.6)
Sex:	Men	3.7 (0.3, 7.1)	2.2 (0.0, 8.9)	2.7 (1.8, 3.7)	6.7 (3.1, 10.4)
	Women	2.2(2.1, 2.2)	0.3 (0.05, 0.5)	1.9 (1.3, 2.4)	4.9 (3.0, 6.9)
Area:	Urban	4.4 (4.3, 4.5)	0.9 (0.0, 3.3)	3.9 (3.6, 4.3)	8.5 (1.6, 15.4)
	Rural	1.7 (0.0, 4.3)	1.2 (0.0, 5.4)	1.0 (0.1, 1.9)	3.9 (1.6, 6.2)
**Newly diagnosed diabetes (n = 654)**					
All		18.9 (15.9, 21.9)	15.2 (7.2, 23.3)	18.8 (12.8, 24.8)	22.9 (16.0, 29.8)
Sex:	Men	20.3 (18.7, 21.9)	19.3 (14.4, 24.3)	20.2 (13.5, 26.9)	21.4 (11.6, 31.1)
	Women	17.9 (13.6, 22.2)	12.3 (1.7, 22.9)	17.7 (12.4, 23.1)	24.3 (9.6, 39.1)
Area:	Urban	20.8 (17.1, 24.6)	14.8 (0.0, 30.4)	20.8 (13.1,28.4)	25.9 (6.3, 45.5)
	Rural	17.6 (13.2, 22.1)	15.5 (7.0, 23.9)	17.3 (8.1, 26.4)	20.8 (18.4, 23.1)
**Confirmed diabetes (n = 292)**					
All		8.1 (5.4, 10.7)	5.8 (3.0, 8.7)	7.8 (4.1, 11.4)	10.9 (6.7, 15.2)
Sex:	Men	9.6 (6.1, 13.1)	8.1 (0.0, 18.0)	9.4 (5.8, 13.0)	11.3 (7.9, 14.7)
	Women	6.9 (4.7,9.0)	4.2 (0.9, 7.4)	6.6 (2.9, 10.3)	10.6 (4.7, 16.6)
Area:	Urban	11.8 (6.4, 17.2)	8.5 (2.4, 14.5)	11.3 (3.3, 19.3)	15.7 (0.7, 30.7)
	Rural	5.5 (3.0, 8.0)	4.4 (2.9, 5.8)	5.2 (4.0, 6.4)	7.5 (4.1, 11.0)
**All diabetes (n = 753)**					
All		21.7 (17.4, 26.1)	16.3 (9.1, 23.5)	21.0 (14.6, 27.4)	28.7 (20.0, 37.3)
Sex:	Men	24.0 (19.6, 28.3)	21.5 (11.8, 31.2)	23.0 (15.5, 30.5)	28.1 (19.5, 36.7)
	Women	20.0 (15.7, 24.3)	12.5 (2.1, 23.0)	19.6 (14.2, 25.0)	29.2 (14.9, 43.6)
Area:	Urban	25.2 (21.6, 28.8)	15.7 (0.0, 33.8)	24.7 (16.7,32.7)	34.4 (7.9, 60.8)
	Rural	19.4 (12.4, 26.4)	16.7 (12.4, 20.8)	18.3 (8.3, 28.3)	24.6 (19.9, 29.3)

* Refers to residents with normoglycaemia and normal HbA1c levels.

As for pre-diabetes, older age was significantly associated with a higher prevalence (*p* = 0.027). There was no difference between men and women (*p* = 0.94), but the rural population had a higher prevalence than the urban population, with borderline significance (*p* = 0.068).

As for diabetic residents, men generally had a higher prevalence than women (*p* = 0.013 for diagnosed diabetes, *p* = 0.0024 for newly diagnosed diabetes, *p*<0.001 for confirmed diabetes, and *p*<0.001 for all diabetes), and the urban population had a higher prevalence than those in the rural region (*p* = 0.006 for diagnosed diabetes, *p* = 0.021 for newly diagnosed diabetes, *p*<0.001 for confirmed diabetes, and *p* = 0.0028 for all diabetes). Older age was associated with a higher prevalence of diabetes (*p* values ranged from <0.001 to 0.013).

**Figure 1:**
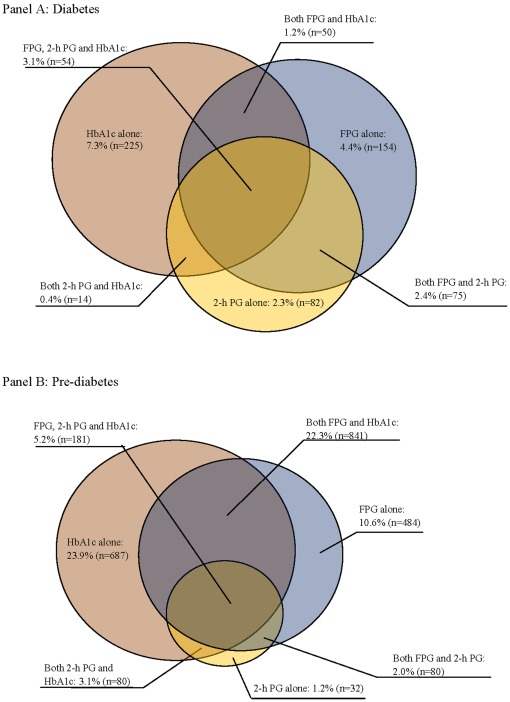
The prevalence of diabetes and pre-diabetes using different screening methods.

Figure 1 shows discordance in the prevalence of newly diagnosed diabetes and pre-diabetes as determined using different diagnostic approaches. The largest proportion of diabetes and pre-diabetes were determined by HbA1c measurement. For the 654 newly diagnosed diabetes patients, 311 were diagnosed by glucose levels (FPG, and/or 2-h PG), 225 were diagnosed by HbA1c measurement, and 118 were diagnosed by both. Only 54 diabetes patients (3.1% of the population) simultaneously had a FPG of ≥7.0 mmol/l, a 2-h PG of ≥11.1 mmol/l, and an HbA1c level of ≥6.5%. For the 2385 pre-diabetes, 596 were diagnosed by glucose levels (FPG, and/or 2-h PG), 687 were diagnosed by HbA1c measurement, and 1102 were diagnosed by both. Only 181 pre-diabetes (5.2% of the population) simultaneously had an IFG, an IGT, and an HbA1c level between 5.7 to 6.4%.

Table 3 shows the comparison of vascular risk factors for diabetes determined by glucose and HbA1c levels. Compared to the diabetes by glucose, patients by HbA1c were generally older, and had higher BMI, waist circumference, blood pressure, lipids (except for HDL-cholesterol) and insulin levels. Similar results were observed for pre-diabetes (Table 3). Table 4 presents the prevalence of diabetes in the GNHS 2002 and the GHS 2010 using different diagnostic approaches. For the GNHS 2002, the prevalence of diabetes by self-reported diagnosis, self-reported diagnosis/FPG test, and self-reported diagnosis/FPG test/2-h PG test for those with an IFG were 1.4%, 2.9% and 2.9%, respectively. For the GHS 2010, the corresponding figures were 2.8%, 11.9%, and 13.8% respectively. If all participants had 2-h PG test, the prevalence of diabetes was 14.6%. The prevalence of diabetes increased to 21.7% if all methods were used for diagnosis (i.e., self-reported diagnosis, FPG test, 2-h PG test, and HbA1c measurement).

**Table 3:** Vascular risk characteristics of diabetes and pre-diabetes determined by glucose and HbA1c levels in the southern Chinese adults 18 years of age or above, 2010.

	Diabetes			Pre-diabetes		
	FPG and/or 2-h PG	HbA1c	*p*	FPG and/or 2-h PG	HbA1c	*p*
	(n = 311)	(n = 225)		(n = 596)	(n = 687)	
Age (years)	48.7 (39.5, 57.9)	55.5 (52.0, 58.9)	0.018	47.5 (43.1, 51.9)	50.0 (40.9, 59.5)	<0.001
Sex (male %)	46.2 (38.8, 53.6)	43.9 (38.8, 49.0)	0.42	44.6 (41.7, 47.6)	42.2 (31.2, 53.4)	0.47
Body mass index (kg/m^2^)	23.4 (19.0, 27.9)	25.4 (20.1, 30.7)	<0.001	23.1 (20.4, 25.8)	23.5 (20.7, 26.3)	0.0003
Waist circumference (cm)	79.4 (71.1, 87.7)	84.5 (75.5, 93.6)	<0.001	75.2 (69.7, 80.6)	77.0 (70.3, 83.6)	<0.001
Systolic blood pressure (mmHg)	103 (98, 109)	109 (104, 115)	<0.001	101 (91, 111)	103 (93, 113)	<0.001
Diastolic blood pressure (mmHg)	75 (74, 77)	79 (76, 81)	<0.001	74 (65, 82)	75 (67, 83)	<0.001
Total cholesterol (mmol/L)	4.17 (3.62, 4.72)	4.59 (4.05, 5.14)	<0.001	3.24 (2.59, 3.88)	3.76 (3.09, 4.42)	<0.001
Triglyceride (mmol/L)	1.53 (0.64, 2.42)	2.01 (1.29, 2.74)	0.0041	0.85 (0.51, 1.20)	1.09 (0.70, 1.49)	0.0068
LDL-triglyceride (mmol/L)	2.14 (1.77, 2.51)	2.46 (2.16, 2.76)	<0.001	1.71 (1.25, 2.17)	2.06 (1.59, 2.53)	<0.001
HDL-cholesterol (mmol/L)	1.08 (0.89, 1.26)	1.00 (0.81, 1.19)	<0.001	0.96 (0.77, 1.16)	1.00 (0.88, 1.13)	<0.001
Fasting insulin (mmol/L)*	7.60 (6.05, 9.55)	8.71 (6.77, 11.21)	<0.001	6.53 (5.67, 7.50)	7.99 (6.91, 9.24)	<0.001
2-hour post-load insulin (mmol/L)*	23.10 (7.32, 72.96)	29.89 (12.48, 71.58)	0.0011	21.79 (18.62, 25.50)	27.35 (18.92, 39.53)	<0.001
HOMA-IR levels*	2.60 (2.23, 3.03)	2.04 (1.53, 2.72)	<0.001	1.72 (1.37, 2.15)	1.78 (1.43, 2.21)	0.0080
Fasting plasma glucose (mmol/L)	8.16 (6.60, 9.73)	5.34 (4.79, 5.88)	<0.001	5.92 (5.25, 6.58)	5.04 (4.28, 5.80)	<0.001
2-hour post-load plasma glucose (mmol/L)	11.87 (8.00, 15.73)	5.61 (4.20, 7.02)	0.024	6.15 (5.50, 6.81)	5.12 (4.13, 6.11)	<0.001
HbA1c levels (%)	5.99 (5.34, 6.65)	7.58 (6.29, 8.88)	<0.001	5.28 (5.19, 5.37)	5.92 (5.81, 6.04)	<0.001

All variables were adjusted by age except for age and sex.

* geometric means.

**Table 4:** The comparison of the prevalence of diabetes in 2002 and 2010 in the southern Chinese adults 18 years of age and above.

Screening methods	The GNHS 2002		The GHS 2010	
	n	Diabetes	n	Diabetes
Self-reporting	102	1.4 (1.1, 1.8)	99	2.8 (1.3, 4.3)
Self-reporting/FPG *	193	2.9 (2.0, 3.7)	432	11.9 (6.2, 17.7)
Self-reporting/FG/partly 2-h PG †	193	2.9 (2.0, 3.7)	501	13.8 (10.2, 17.3)
Self-reporting/FG/all 2-h PG ‡	-	-	528	14.6 (13.3, 15.9)
Self-reporting/FG/2-h PG/HbA1c ||||	-	-	753	21.7 (17.3, 26.1)

* : diabetes was diagnosed by self-reporting in an interview and with fasting plasma glucose (FPG) test.

† : diabetes was diagnosed by self-reporting in an interview, with FPG test and with 2-hour post-load plasma glucose (2-h PG) test for those with an IFG.

‡ : diabetes was diagnosed by self-reporting in an interview, with FPG test and with 2-h PG test for all participants.

||||: diabetes was diagnosed by self-reporting in an interview, with FPG, with 2-h PG test and with HbA1c measurement.

## Discussion

The results of the representative, population-based GHS 2010 show that the prevalence of diabetes based on glucose levels and the HbA1c criterion is alarmingly high at 21.7% in this developing southern Chinese population, which suggests that more than 1 in 5 adults 18 years of age and above are suffering from diabetes. This translates to a total of 13.1 million adults having diabetes in the Guangdong province, which has a population of 85 million residents. This is in conjunction with an extremely low awareness of diabetes in this region, with only 12.9% diabetic residents knowing that they had the condition. The prevalence of pre-diabetes in this population was 66.3%, which translates to approximately 40.0 million adult residents who are at risk of diabetes and cardiovascular disease. Our results also suggest that the glucose test and the HbA1c measurement taken together can significantly increase the detection of diabetes and pre-diabetes among Chinese residents. Using only one single method to screen and diagnose diabetes in large general populations is likely underestimate the diabetic situation.

The prevalence of diabetes in China was relatively low before the year 2000 but has increased dramatically in last decade. In 1994, a national survey of the Chinese population between 25 to 64 years of age showed that the prevalence of diabetes and IFG was 2.5% and 3.2%, respectively [5]. Gu et al. conducted the International Collaborative Study of Cardiovascular Disease in Asia in 2001, and their results show that the prevalence of diabetes and IFG among Chinese people between 35 to 74 years of age was 5.5% and 7.3%, respectively [6]. The results of the National Nutrition and Health Survey 2002 (NNHS 2002) showed that the national prevalence of diabetes and IFG was 2.7% and 4.9% for Chinese people 20 years of age or above, respectively [8]. However, the age-standardised prevalence of diabetes in large cities reported in the NNHS 2002 was 7.8%, which was already close to the prevalence of 9.3% reported in the NHANES 1999–2002 in the U.S. [8], [18]. The results from the survey by Yang et al. conducted in 2007 showed that the prevalence of diabetes and IFG reached 9.7% and 15.5% nationwide [7]. All of these studies applied the glucose criteria for the diagnosis of diabetes and IFG, but only the study by Yang et al. applied the 2-h PG test to all participants. In the present study, the FPG test and the 2-h PG test were applied to all participants. The prevalence of diabetes and IFG based on FPG and 2-h PG were 14.6% and 25.3%, respectively. It is astonishing that the prevalence of diabetes based on the same diagnostic method increased from 2.9% in 2002 to 13.8% in 2010 in this southern Chinese population (Table 4). Furthermore, the prevalence of diabetes and pre-diabetes based on the glucose and HbA1c criteria in the present study were 21.7% and 66.3%, respectively. Though our sample is only representative for the southern Chinese population of 85 million residents in the GHS 2010, these results suggest that the diabetes epidemic is accelerating in China.

The prevalence of pre-diabetes in this population is trembling. Approximately 40.0 million adult residents have at least one of the following three conditions: IFG, IGT, or an HbA1c level between 5.7%–6.4%. Studies have shown that 25%–40% individuals with IFG or IGT will develop diabetes over the next 3 to 8 years [19], [20]. The HbA1c level was a strong predictor of subsequent diabetes and cardiovascular events [21], and individuals with an HbA1c value between 5.5% and 6.5% had a substantial increased risk for developing diabetes within 5 years [22], [23].

Rapid economic development and urbanization, an aging population and a lifestyle transition with a consequent obesity epidemic are likely the major contributors to the astonishing increase of diabetes and pre-diabetes in this population in last decade [24]. The data from our GNHS 2002 study show that the mean age of the adult residents 18 years of age or above in this population was 44.1 years, while it was 50.1 years in the GHS 2010. The prevalence of obesity, especially central obesity, increased dramatically in the last decade. The mean waist circumference was 75.0 cm in the year 2002, while it was 79.8 cm in the year 2010. Furthermore, the increased magnitudes are more prominent in the rural regions than those in the urban regions. In 2002, the mean waist circumference was 73.1 cm for the rural population and 77.0 cm for the urban population, while in 2010, the corresponding figures increased to 79.7 cm and 79.9 cm, respectively. Similar changes were also observed for other risk factors, including BMI, lipids and blood pressure (data not shown here). Our results have shown that the rural population had a higher prevalence of pre-diabetes than the urban population (Table 2). In addition to the dramatic increase in vascular risk, studies have shown that the Asian populations have a higher prevalence of diabetes and are more likely to develop diabetes earlier compared to the white Caucasian population, despite the fact that Asians have a smaller body mass index [25], [26], [27]. This may also partly explain the steep increase in diabetes in our population in last decade.

The high prevalence of diabetes and pre-diabetes in the present study are also related to the thorough investigation using the glucose and HbA1c tests together. The FPG test plus the OGTT is currently the most common approach for screening diabetes in the general population. However, the 1999 WHO guidelines only recommend the OGTT for those patients with IFG. Our GHNS 2002 study showed that the FPG plus the 2-h PG test, restricted for those people with IFG, did not increase the detection rate of diabetes (Table 4), as the prevalence of IFG was low in the population in the year 2002, and only 106 participants received the 2-h PG test, which resulted in little increase in the detection rate of diabetes. In the present study, the 2-h PG test restricted to those people with IFG diagnosed an additional 1.9% of residents as diabetic. The 2-hour PG test applied for those participants without IFG diagnosed additional 0.8% residents as diabetic (Table 4). In previous surveys, the 2-hour PG test was generally applied to those participants with IFG for screening diabetes except in the study by Yang et al. [5], [6], [7], [8]. These surveys might underestimate the prevalence of diabetes.

Until 2010, the diagnosis of diabetes was based solely on glucose concentration. In contrast to glucose measurement, the HbA1c test does not require a fasting sample; thus, it is more convenient. However, the HbA1c criteria are relatively new, and there are still concerns, including the standardised HbA1c measurement, a cut-off point, different ethnic distributions and a discordance with the glucose criteria [28], [29], [30], which could encumber the wide use of the HbA1c test for diabetes screening and diagnosis. In our GHS 2010, when we applied the cut-off point of 6.3% as suggested by Bao et al. for the Chinese population [31], the prevalence of diabetes increased to 28.2%. Nonetheless, due to the proven relationship of HbA1c levels with diabetes-related complications and vascular diseases, a large number of people will be missed without the HbA1c measurement even though they are suffering from diabetes or at risk of diabetes and cardiovascular disease, which means they need treatment and care. Our results show that diabetes and pre-diabetes determined by HbA1c had higher vascular risk than those determined by glucose levels (Table 3). In our GHS 2010, based on the cut-off value of 6.5% recommended by the ADA, the HbA1c criterion alone diagnosed 12.0% of residents as diabetic (Figure 1) and added 7.1% residents as diabetic, in addition to the patients diagnosed by the glucose criteria and self-reporting (Table 4). Our results support the use of both the glucose test and the HbA1c test together for diabetes screening and diagnosis [23], [30].

The strengths of our study include a representative sample that targeted a large southern Chinese population of 85 million residents and a thorough investigation on diabetic conditions using all three screening and diagnosis approaches, including the FPG test, the 2-h PG test and the standardised HbA1c measurement in a NGSP-certified laboratory. We also assessed the diabetes trend in this population by comparing the data from the GNHS 2002. One limitation is that each test (the FPG, the 2-h PG and the HbA1c) was performed once for each participant in the present study, which limited us when calculating the accurate prevalence of confirmed diabetes. Based on the ADA criteria, retesting is suggested to confirm a diagnosis in a clinical setting if diabetes is determined by one single test [14].

In summary, the prevalence of diabetes and pre-diabetes in the Guangdong province as determined by the HbA1c test and the glucose test was alarmingly high at 21.7% and 66.3%, respectively. We estimate that a total of 13.1 million adult residents have diabetes and that 40.0 million adult residents are at risk of developing diabetes and cardio-vascular disease. The prevalence of diabetes and pre-diabetes has increased dramatically within an 8-year period in the Guangdong province with rapid economic development, which suggests that the diabetes epidemic is accelerating in China. Our results suggest that the glucose and the HbA1c approaches should be used together for the screening and diagnosing of diabetes and pre-diabetes. One single approach will underestimate the prevalence of diabetes and miss a substantial proportion of the diabetic patients or adults at risk for diabetes and vascular disease, who may require treatment and care. The awareness of diabetes in this developing population is extremely low. Urgent public health actions are needed to control this worsening situation in China.
